# Surgical techniques for evacuation of chronic subdural hematoma: a mini-review

**DOI:** 10.3389/fneur.2023.1086645

**Published:** 2023-06-28

**Authors:** Benjamin Rodriguez, Isabella Morgan, Tirone Young, Joseph Vlastos, Tyree Williams, Eugene I. Hrabarchuk, Jaden Tepper, Turner Baker, Christopher P. Kellner, Joshua Bederson, Benjamin I. Rapoport

**Affiliations:** ^1^Mount Sinai BioDesign, Mount Sinai Medical System, New York, NY, United States; ^2^Department of Neurosurgery, Mount Sinai Medical System, New York, NY, United States; ^3^Icahn School of Medicine, New York, NY, United States; ^4^Rensselaer Polytechnic Institute, Troy, NY, United States

**Keywords:** surgical techinque, burr hole and drainage, endoscope assistance, MMA embolization, twist drill craniostomy, craniotomy, subdural evacuating port system (SEPS)

## Abstract

Chronic subdural hematoma is one of the most common neurosurgical pathologies with over 160,000 cases in the United States and Europe each year. The current standard of care involves surgically evacuating the hematoma through a cranial opening, however, varied patient risk profiles, a significant recurrence rate, and increasing financial burden have sparked innovation in the field. This mini-review provides a brief overview of currently used evacuation techniques, including emerging adjuncts such as endoscopic assistance and middle meningeal artery embolization. This review synthesizes the body of available evidence on efficacy and risk profiles for each critical aspect of surgical technique in cSDH evacuation and provides insight into trends in the field and promising new technologies.

## Introduction

Chronic subdural hematoma (cSDH) is a collection of fluid, blood, and blood degradation products positioned between the dura mater and the arachnoid linings on the brain surface (1, 2). This condition has a compressive effect on the brain leading to neurological deficits that depend on the size and location of the hematoma. The incidence of cSDH is 8.2–14.0 per 100,000 people annually worldwide and is most common in patients over age 70 (3–11). Due to the aging of the population, the incidence of cSDH is anticipated to double by 2,037 (12, 13).

It was previously postulated that all forms of SDH were caused by venous bleeding from bridging veins (draining from the cortical surface into the dura) leading to the accumulation of blood in the subdural space (14, 15). This acute accumulation leads to clot formation, while persistent clots lead to the formation of fibrous membranes. These membranes form their own microvasculature over time through neo-angiogenesis, and bleeding from these small blood vessels contributes to further expansion, persistence, and recurrence of chronic subdural hematomas (16–18). Like the initial bleeding, rebleeding usually occurs within the inner capillary layer of the dura (15, 19–22). These capillaries are distal branches of the middle meningeal artery (MMA) (17, 23).

**Figure 1:**
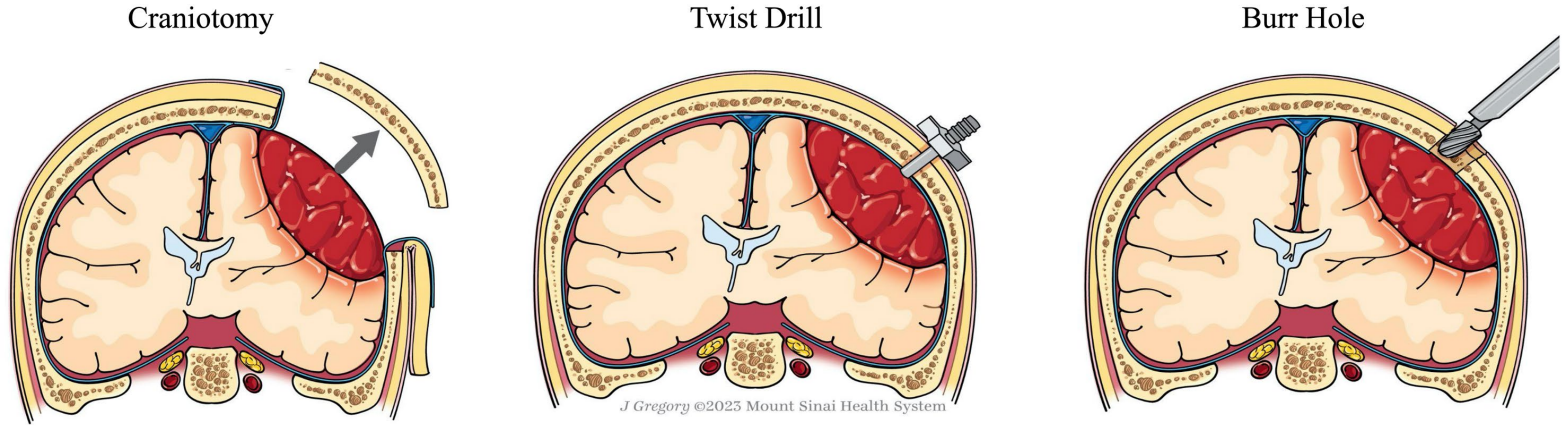
Illustration of the three evacuation techniques.

Standard treatment for cSDH involves evacuation of the hematoma to reduce the mass effect and alleviate symptoms (Figure 1) (12). Spontaneous resolution of significant thickness has been reported in a small number of case series, however, it is generally accepted that in the presence of focal symptoms and/or changes in neurologic status, patients should undergo immediate surgical evacuation (12, 24). Effective evacuation improves patient outcomes and reduces the likelihood of recurrence (25).

In the past decade, there has been an increase in the utilization of neuroendoscopy to provide enhanced visualization during hematoma evacuations. Surgical treatment of intracerebral hemorrhage, for example, has seen a recent growth in available tools to enable hematoma evacuation under direct visualization (26, 27). With case studies of neuroendoscopic SDH evacuation now beginning to appear in the literature (28, 29), it is important for the field to stay current on the alternative techniques for cranial access, evacuation, and postoperative drainage, as well as how these techniques may interact with the development and adoption of more modern strategies.

This mini-review aims to concisely summarize historically utilized evacuation techniques for cSDH while contextualizing the modern development of neuroendoscopic SDH evacuation as well as other adjunct procedures.

## Methods

A literature search was conducted across three major databases (PubMed, Elsevier, Google Scholar) using the terms “evacuation of chronic subdural hematoma” AND (“minimally invasive” OR “bedside” OR “SEPS” OR “burr hole” OR “craniotomy” OR “twist drill” OR “endoscopy”) AND “elderly.” Additional studies were identified from the references of previously published reviews and included in the analysis.

Publications reporting surgical treatment of acute subdural hematoma (aSDH) were not included given the large difference in pathogenesis, hematoma consistency, and surgical intervention strategies (Figure 1).

### Evacuation techniques

#### Twist drill craniostomy

Twist drill craniostomy is the most minimally invasive surgical technique for SDH evacuation, wherein a standard bedside twist drill creates a small (<5 mm) burr hole. The dura is incised and a cannula accesses and passively drains the hematoma. The procedure is commonly performed at the bedside using only local anesthesia (5, 30, 31).

The primary benefits of this technique are reduced invasiveness and the avoidance of general anesthesia, resulting in a lower overall procedural risk, particularly in the elderly or in patients with medical comorbidities (32–34). Reported twist drill craniostomy morbidity ranges from 2.5 to 4.4% and mortality ranges from 2.9–5.1% (12, 30, 35); both significantly lower than other techniques. This lower risk profile comes at the cost of effective treatment, with twist drill craniostomy demonstrating evacuation rates significantly lower than other techniques and resulting in a corresponding higher recurrence rate ranging from 28.1–31.3% (12, 30, 35).

Twist drill craniostomy is the only technique that has seen the development of specialized tools exclusively for the treatment of SDH. These systems, called negative pressure (NPE) evacuation systems, include a stainless steel port that is inserted through the burr hole and connected to a bulb suction reservoir that applies negative pressure and actively drains the hematoma (5, 36–38). Safain et al. compared burr hole and NPE in balanced cohorts of 23 patients and found no significant difference in mortality (4.3% for NPE, 9.1% for burr hole evacuation) or length of stay (11 days for NPE, 9.1 days for burr hole evacuation) between the two groups (39). Furthermore, multiple studies have concluded that the efficacy of NPE is comparable to that of twist drill or burr hole evacuation (9, 30, 40, 41). Since their development, these systems have seen widespread adoption across the field, with Singla et al. reporting that the number of SEPS (Medtronic, United States) devices ordered in the USA increased almost threefold from 2007 to 2011 (42). Their popularity can be attributed to their minimal invasiveness and ability to be performed at the bedside, which is more resource efficient for the hospital system and the patient.

#### Burr hole craniostomy

Burr hole craniostomy (BHC) is the most common technique for SDH evacuation. BHC begins by drilling one or two 12–14 mm burr holes on the cerebral convexity approximately 5–8 cm apart (40, 43–45). The dura is incised and the hematoma is evacuated using a combination of suction and irrigation (46). Normal saline (NS) and artificial cerebrospinal fluid (ACSF) are commonly used as irrigation solutions, with NS being the most common. Recent studies indicate that ACSF, as an irrigation solution, may improve treatment effectiveness. A study including 234 consecutive patients by Kuwabara et al., found a 23.8% recurrence in patients treated with NS, and a 9.0% recurrence in patients that were treated with ACSF as an irrigation solution (47). Additionally, a retrospective study by Bartley et al. found that irrigating the solution at body temperature results in lower recurrence rates than at room temperature (48). BHC is most commonly performed under general anesthesia, though local anesthesia is a feasible alternative, with one recent study finding local anesthesia evacuation to result in significantly lower complication rates compared to BHC evacuations performed under general anesthesia (49, 50).

Similar to twist drill craniostomies, this technique is preferred in the elderly population, where the increased trauma associated with craniotomy has deleterious effects (51–53). Morbidity and mortality rates in BHC cases remain low, ranging from 4–9.3% and 2.5–3.7%, respectively. Recurrence rate is reported to range from 10.5 to 12.0%, significantly lower than twist drill craniostomy recurrence rates (5, 12, 30, 54).

No specialized tools have yet been developed exclusively for the evacuation of SDH utilizing the burr hole craniostomy technique.

#### Craniotomy

Craniotomy is the most invasive, but the most surgically effective technique for evacuating cSDH. A bone flat bone varying from 3 to 5 cm in diameter or larger is elevated over the cSDH and the dura is incised in a cruciate fashion to evacuate the hematoma and allow fluid to drain out of the subdural space. The hematoma membranes can be coagulated and excised to prevent further bleeding. Once the hematoma is completely evacuated, the surgical field is inspected to ensure complete hemostasis, and then the dura can be reapproximated either primarily (using sutures) or with the assistance of dural substitute to facilitate secondary healing. The skull flap is then replaced (51). The procedure is performed in the operating room under general anesthesia (5, 31). Morbidity and mortality associated with this approach were 4–12% and 4.6–12.2%, respectively (5, 12, 30, 35, 54). However, this low morbidity and mortality rate may be due to selection bias, with physicians opting for alternative techniques (such as burr hole evacuation) in the elderly and other fragile populations. The recurrence rate is reported to range from 11 to 19.4% (12, 30).

No specialized tools have yet been developed exclusively for the evacuation of SDH utilizing craniotomy access.

### Promising adjunct therapies

#### Drainage

Following the evacuation, the surgeon has the opportunity to position a surgical drain within the subdural or subgaleal space. Leaving a drain in place for 48 h postoperatively has been found to significantly reduce the risk of symptomatic recurrence and the need for reoperations (36, 46, 55–58). Postoperative drainage can be used in conjunction with any surgical evacuation technique, but it has been most extensively studied in conjunction with burr hole drainage. Several studies have demonstrated that 24–48 h of postoperative drainage leads to improved outcomes relative to no drainage. The optimal duration of postoperative drainage remains an open question, however (30, 40, 59–63). Jensen et al. for example, reported no significant differences in the rate of recurrence or death during 90-day follow-up between groups that received either 48-h or 24-h of passive drainage after burr hole evacuation of cSDH (63).

Optimal drain location remains an open question, with no clear advantage in recurrence rates between subdural or subgaleal drain placement (64). It has been suggested that in the absence of a difference in efficacy, subgaleal drain placement should be preferred on the basis of relative safety over subdural drains (65).

#### Endoscopic assistance

The use of neuroendoscopy allows for enhanced visualization as the hematoma is evacuated, enabling visualization of trabeculae and septations even when employing burr hole evacuation techniques. This visualization facilitates more complete hematoma evacuation as well as excision of neomembranes and meticulous microscopic hemostasis (66, 67). Neuroendoscopic techniques have been reported in both craniotomy (68) and burr hole evacuations (28, 67, 69–73). Data from early studies suggest that the use of neuroendoscopy in burr hole evacuations results in low complication rates, and reduced recurrence rates (28, 74, 75). Both rigid and flexible endoscopes have been studied for SDH evacuation, with a majority of the studies using rigid endoscopes. Rigid endoscopes are generally preferred due to their availability, image quality, and versatile sheaths with working channels for functions such as irrigation, suction, grasping forceps, and coagulation. In their 25-patient study, Huang et al. mention opting for a rigid endoscope for such reasons (29). No twist drill neuroendoscopic cases were identified, likely due to the access size being too small for current endoscope technologies.

#### Middle meningeal artery embolization

Middle meningeal artery (MMA) embolization is a modern intervention in which embolization material is delivered to the subdural neomembrane capillaries through catheter-based endovascular techniques. These small vessels are thought to be responsible for expansion and recurrence, and the intent of embolizing them is to restrict blood flow to the subdural neomembranes and thereby inhibit hematoma expansion and recurrence (17, 76–78).

Several cohort studies have suggested that MMA embolization in conjunction with evacuation reduces recurrence rates and is associated with low procedural complication rates (79–89). The embolic agents being studied include the SQUID embolic agent, the ONYX liquid embolic system and, TRUFILL n-BCA. These results have not yet been established in a randomized trial, however, several such trials are ongoing (90–92). The efficacy of MMA embolization as a stand-alone treatment for cSDH without surgical evacuation is similarly being investigated in one arm of the MEMBRANE trial (91).

Ironside et al. found in a meta-analysis of 20 studies comprising 1,416 patients that MMA embolization was performed up-front (as a stand-alone therapy, without evacuation) in 28.4% of patients, as a post-surgical adjunct in 23.2%, and as a rescue therapy following cSDH recurrence in 47.8% (77). Onyinzo et al. described a similar distribution of patients in a 132-patient study.

Since its first description in 2000 by Mandai, evidence has accumulated suggesting that MMA embolization is beneficial when added to standard-of-care treatment for cSDH (80–89, 93). A multicenter study published by Kan et al. evaluated 154 MMA embolizations performed not as adjunct treatment (94). Only 9 patients required a second (salvage) intervention (6.5%) and the thickness of the cSDH was improved in 140 patients (90.9%). General anesthesia was used in 6.1% of these patients.

A meta-analysis by Jumah et al. of 177 patients in 11 studies reported a treatment failure rate of 2.8%, an embolization complication rate of 1.2%, and a surgical rescue rate of 2.7% (95). This demonstrates that MMA embolization can be an effective adjunct therapy alongside surgical evacuation. A 72-patient case series performed by Ban et al. compared outcomes in cSDH patients treated with preoperative MMA embolization as compared with surgical intervention alone (79). Not only was the recurrence rate in the presurgical MMA embolization group significantly lower than the rate following surgery alone (1.4% versus 27.4%), but the embolized group also had a significantly lower rate of surgical complications (0 and 4.3% respectively).

## Discussion

Despite widespread experience with conventional surgical treatments and a considerable amount of clinical research, there remains a need for continued innovation in the space of surgical cSDH evacuation. While the minimally invasive techniques of twist drill craniostomy and burr hole craniostomy enable the treatment of more fragile populations, they suffer from higher recurrence rates due to reduced evacuation percentages. The more invasive craniotomy technique has seen great success in younger populations, but due to high morbidity and mortality rates, it is not optimal for many elderly cSDH patients.

The development of specialized tools and the potential for future combination therapies is an exciting step toward improved patient care. The use of perioperative drains has demonstrated strong clinical evidence to reduce recurrence rates, however, additional research is needed to optimize placement and length of treatment. Furthermore, the negative pressure systems broadly used with twist drill craniostomy have seen success over the past decade, yet new tools should continue to be developed that utilize modern technological advances. The integration of neuroendoscopy into cSDH evacuation has the potential to improve evacuation rates through minimally invasive cranial access, however significant clinical research and the development of specialized systems are still needed. Similarly, more clinical studies are needed to determine the potential of an evacuation-embolization combination therapy (96). A summary of the different characteristics of each treatment and whether the treatment interfaces with an adjunct technique can be found in Table 1.

**Table 1:** Technical breakdown of cSDH evacuation techniques.

	Access Size	Anesthesia	Evacuation Style	Irrigation	Specialized Tools	Adjunct Treatment Capabilities		
						Endoscopy	Postop Drainage	MMA
“Twist Drill Craniostomy”	~5 mm	Local	Passive	No	Negative-Pressure Evacuators	No	Yes	Yes
“Burr Hole Craniostomy”	~14 mm	Local or General	Active	Yes	None	Yes	Yes	Yes
“Craniotomy”	~30 mm +	General	Active	Yes	None	Yes	Yes	Yes

MMA, Middle Meningeal Embolization.

cSDH is expected to become one of the most common neurosurgical procedures in the U.S. as the population ages. It is important for the field to continually assess the state of current techniques and the future potential of new technologies to find optimal procedures to maximize hematoma resection while minimizing procedural invasiveness.

## Conclusion

The purpose of this mini-review is to present the current treatment options used in the surgical management of chronic subdural hematoma and how they interface with emerging adjunct techniques. Each treatment and technique is briefly described, and associated patient outcomes are presented. The patient data were gathered from large patient studies, well-known RTCs, clinical trials, systematic reviews, and meta-analyzes. The surgical treatments were classified by the size of the access aperture, type of anesthesia, type of evacuation, irrigation or specialized instruments are used, and by the potentially available adjunctive options. Adjunct treatment options currently in development were also summarized. Ultimately, more work must be done to define an evidence-based approach to decide among treatment options in a given case, and to develop tools and techniques to reduce hematoma recurrence while minimizing procedural invasiveness.

## Author contributions

BRo primary author, data collection, editing, and figure creation. IM: data collection and editing JV: data collection, editing, and formatting. TW and EH: editing and formatting. JT: Figure creation. TB, BRa, CK, and JB: editing and administrative leadership. All authors contributed to the article and approved the submitted version.

## Conflict of interest

The authors declare that the research was conducted in the absence of any commercial or financial relationships that could be construed as a potential conflict of interest.

## Publisher’s note

All claims expressed in this article are solely those of the authors and do not necessarily represent those of their affiliated organizations, or those of the publisher, the editors and the reviewers. Any product that may be evaluated in this article, or claim that may be made by its manufacturer, is not guaranteed or endorsed by the publisher.
